# A comparison of transmissibility of SARS-CoV-2 variants of concern

**DOI:** 10.1186/s12985-023-02018-x

**Published:** 2023-04-02

**Authors:** S. S. Manathunga, I. A. Abeyagunawardena, S. D. Dharmaratne

**Affiliations:** 1 National Hospital of Sri Lanka, Colombo, Sri Lanka; 2 Department of Community Medicine, Faculty of Medicine, University of Peradeniya, Kandy, Sri Lanka; 3 Department of Health Metrics Sciences, Institute for Health Metrics and Evaluation, School of Medicine, University of Washington, Seattle, USA

**Keywords:** Coronavirus, COVID-19 variants, Transmissibility

## Abstract

**Background:**

The World Health Organization (WHO) has currently detected five Variants of Concern of SARS-CoV-2 having the WHO labels of ‘Alpha’, ‘Beta’, ‘Gamma’, ‘Delta’ and ‘Omicron’. We aimed to assess and compare the transmissibility of the five VOCs in terms of basic reproduction number, time-varying reproduction number and growth rate.

**Methods:**

Publicly available data on the number of analyzed sequences over two-week windows for each country were extracted from covariants.org and GISAID initiative database. The ten countries which reported the highest number of analyzed sequences for each of the five variants were included in the final dataset and was analyzed using R language. The epidemic curves for each variant were estimated utilizing the two-weekly discretized incidence data using local regression (LOESS) models. The basic reproduction number was estimated with the exponential growth rate method. The time-varying reproduction number was calculated for the estimated epidemic curves by the ratio of the number of new infections generated at time step t to the total infectiousness of infected individuals at time t, using the EpiEstim package.

**Results:**

The highest R0 for the variants Alpha (1.22), Beta (1.19), Gamma (1.21), Delta (1.38) and Omicron (1.90) were reported from Japan, Belgium, the United States, France and South Africa, respectively. Nine out of ten epidemic curves with the highest estimated growth rates and reproduction numbers were due to the Omicron variant indicating the highest transmissibility.

**Conclusions:**

The transmissibility was highest in the Omicron variant followed by Delta, Alpha, Gamma and Beta respectively.

## Background

The World Health Organization (WHO) has been monitoring the variants of SARS-CoV-2 to detect the variants that are likely to pose a significantly increased threat to global public health. In collaboration with other researchers and institutes, characterization and classification of the variants was implemented, which helped in prioritizing the monitoring of Variants of Interest (VOI) and Variants of Concern (VOC) [1].

A VOI is defined by the WHO as ‘A SARS-CoV-2 variant with genetic changes that are predicted or known to affect virus characteristics such as transmissibility, disease severity, immune escape, diagnostic or therapeutic escape and identified to cause significant community transmission or multiple coronavirus disease (COVID-19) clusters, in multiple countries with increasing relative prevalence alongside increasing number of cases over time, or other apparent epidemiological impacts to suggest an emerging risk to global public health’. The variants that meet the criteria for VOI, which are also demonstrated to be associated with increase in transmissibility or detrimental change in COVID-19 epidemiology, or increase in virulence or change in clinical disease presentation or, decrease in the effectiveness of public health and social measures or available diagnostics, vaccines, therapeutics are classified as Variants of Concern [1].

There are five Variants of Concern by the time of writing this article, having the WHO labels of ‘Alpha’, ‘Beta’, ‘Gamma’, ‘Delta’ and ‘Omicron’ which have the Pango lineage identifiers of B.1.1.7, B.1.351, P.1, B.1.617.2, B.1.1.529 respectively. Following a critical expert assessment involving the Technical Advisory Group on Virus Evolution of WHO, a variant may be re-classified into a different group at a later stage [2].

We aimed to assess and compare the transmissibility of the five VOCs in terms of basic reproduction number (R0), time-varying reproduction number and growth rate.

## Methods

Publicly available data on the number of analyzed sequences over two-week windows for each country were extracted from covariants.org and Global Initiative on Sharing All Influenza Data (GISAID) initiative databases [3, 4]. Raw data were filtered to contain the incidence data for the five VOCs. The ten countries which reported the highest number of analyzed sequences for each of the five variants were included in the final dataset and was analyzed using R language.

The epidemic curves for each variant were estimated utilizing the two-weekly discretized incidence data using local regression (LOESS) models [5]. The basic reproduction number was estimated with the exponential growth rate method using the R0 package [6, 7]. As Wallinga et al. summarized, the basic reproduction number (R0) can be estimated using the formula

$$R0= \frac{1}{M(-r)}$$

where M is the moment generating function of the discretized generation time interval of the infection and r is the growth rate [8].

The time-varying reproduction number was calculated for the estimated epidemic curves by the ratio of the number of new infections generated at time step t to the total infectiousness of infected individuals at time t, using the EpiEstim package [9, 10].

## Results

**Fig. 1:**
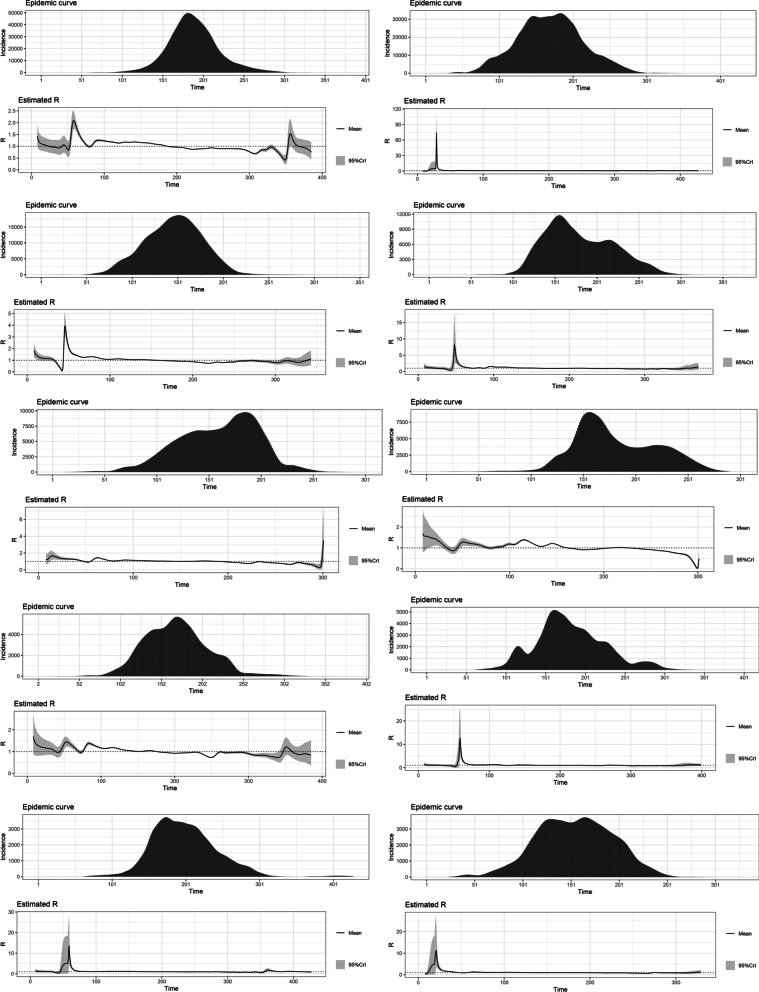
The variations of the estimated time-varying reproduction number of the epidemic curves of the Alpha variant and the uncertainty

**Fig. 2:**
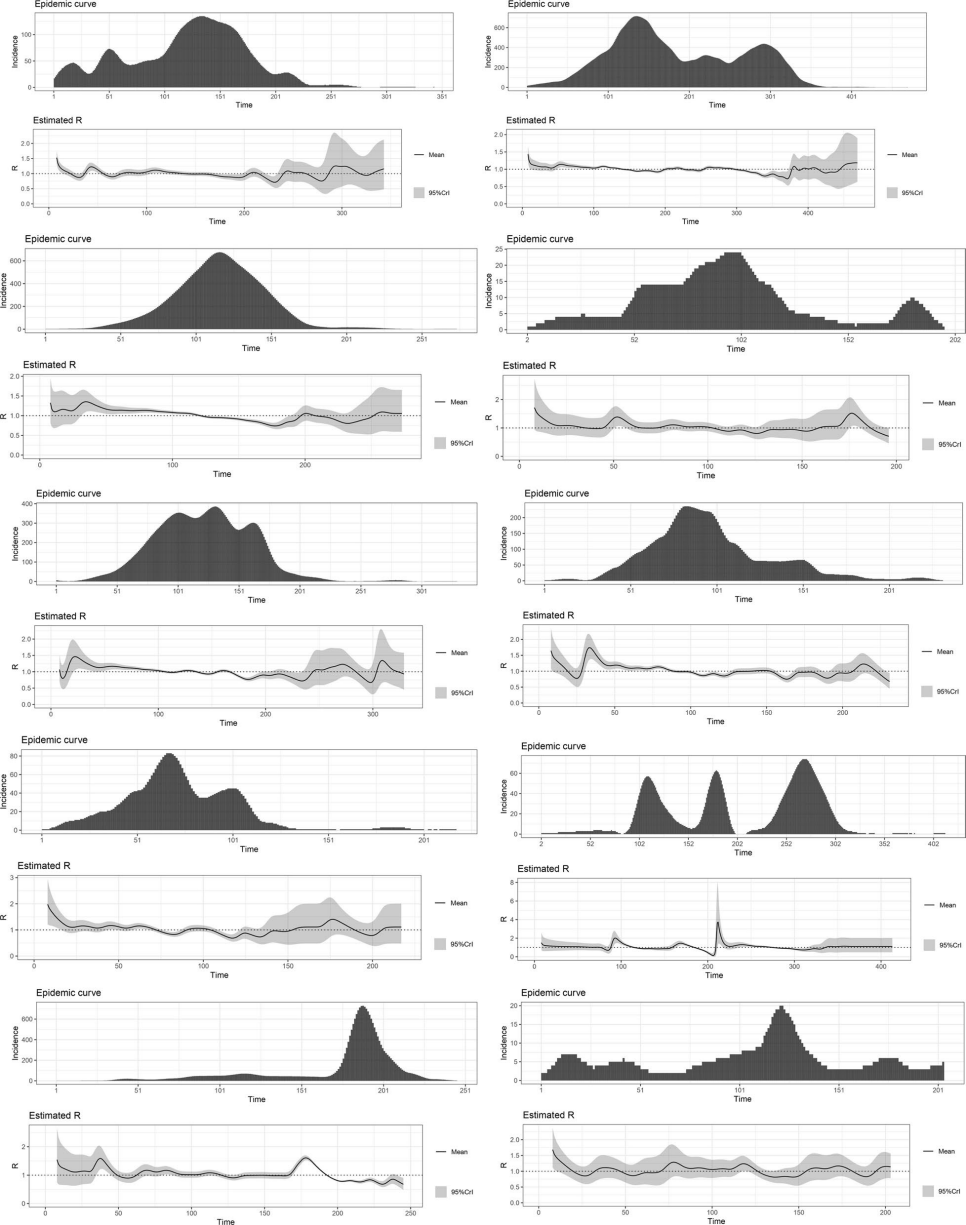
The variations of the estimated time-varying reproduction number of the epidemic curves of the Beta variant and the uncertainty

**Fig. 3:**
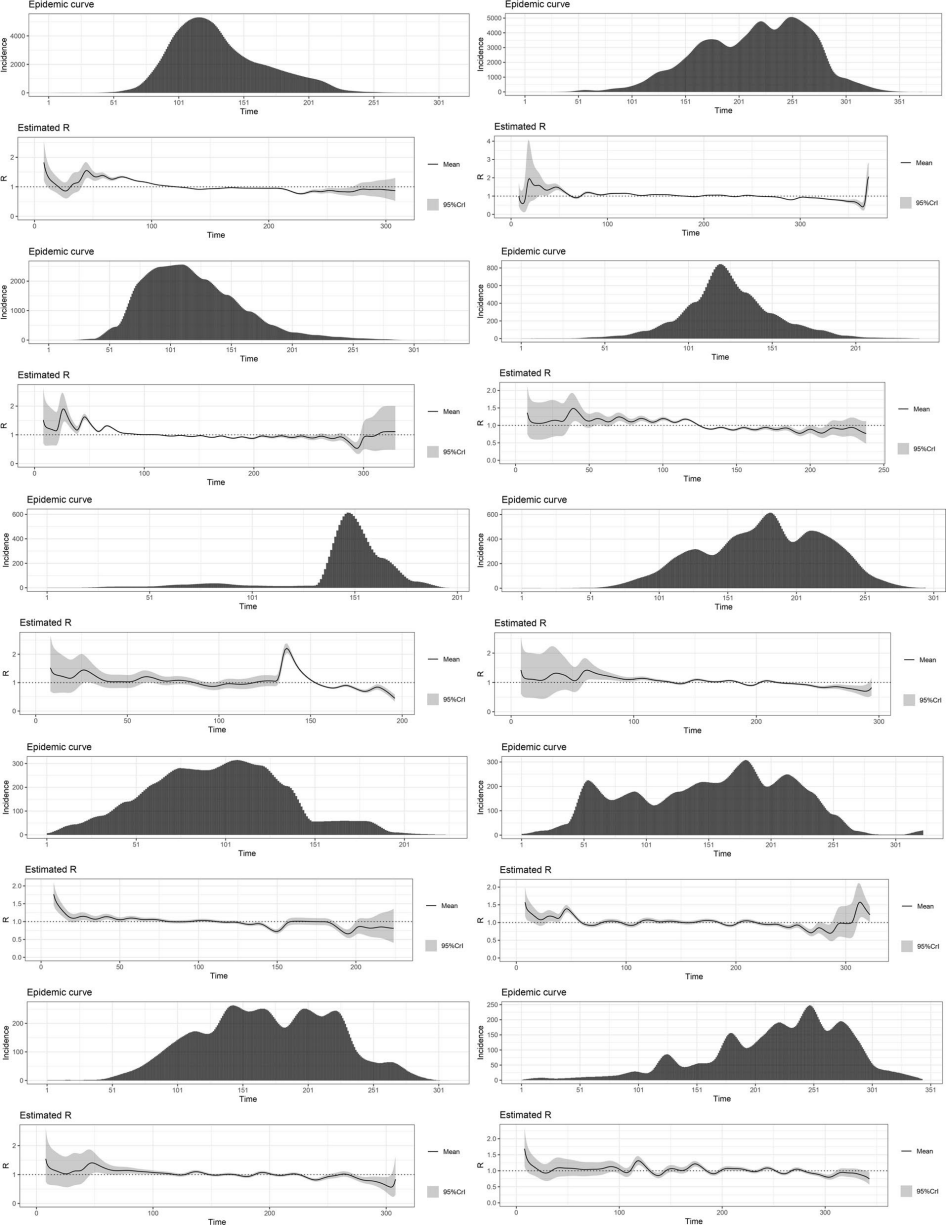
The variations of the estimated time-varying reproduction number of the epidemic curves of the Gamma variant and the uncertainty

**Fig. 4:**
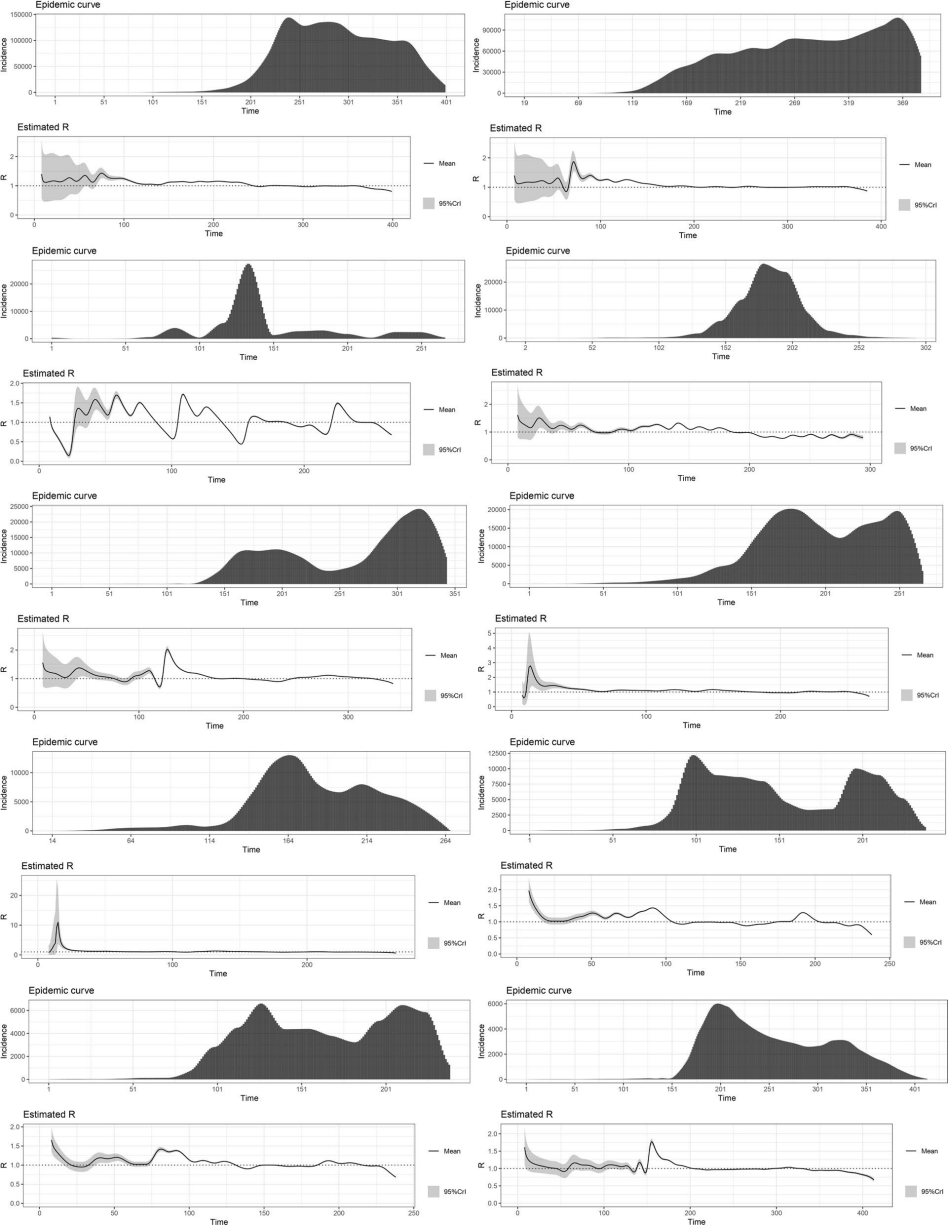
The variations of the estimated time-varying reproduction number of the epidemic curves of the Delta variant and the uncertainty

**Fig. 5:**
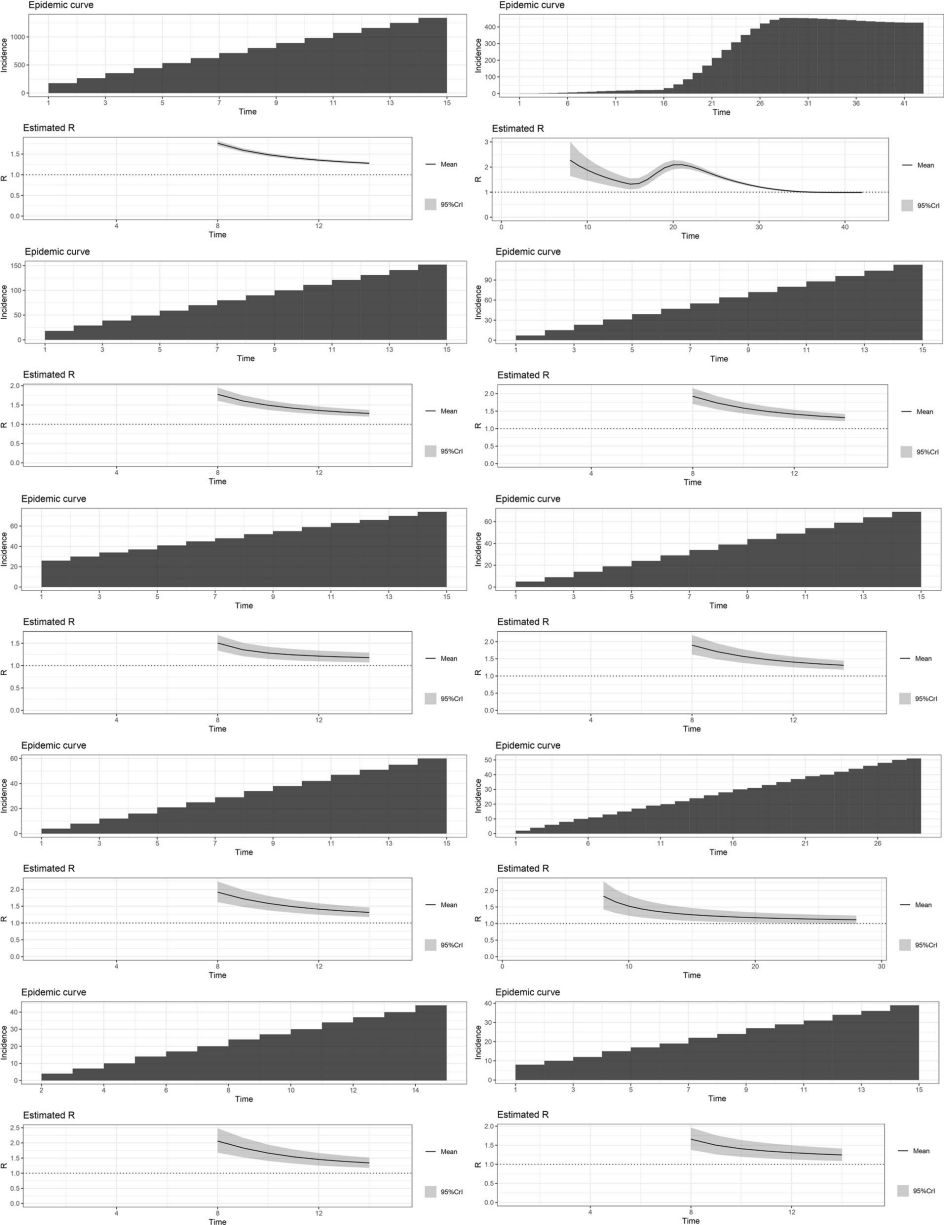
The variations of the estimated time-varying reproduction number of the epidemic curves of the Omicron variant and the uncertainty

The highest R0 for the variants Alpha (1.22), Beta (1.19), Gamma (1.21), Delta (1.38) and Omicron (1.90) were reported from Japan, Belgium, the United States, France and South Africa, respectively. The estimated basic reproduction numbers and growth rates for each variant are tabulated below. The variations of the estimated time-varying reproduction number of the epidemic curves and their uncertainty, in the same order as the table, are depicted below (Figs. 1, 2, 3, 4, 5 and Tables 1, 2, 3, 4, 5).

**Table 1:** Variations of the estimated time-varying reproduction number of the epidemic curves and their uncertainty of the Alpha variant

Country	R	CI lower	CI upper	R squared	Growth rate	Growth rate CI lower	Growth rate CI upper
Japan	1.22	1.22	1.22	0.99	0.06	0.06	0.06
Sweden	1.20	1.20	1.20	0.94	0.05	0.05	0.05
United States	1.19	1.18	1.19	0.99	0.05	0.05	0.05
Italy	1.18	1.18	1.18	0.98	0.05	0.05	0.05
France	1.15	1.15	1.15	0.94	0.04	0.04	0.04
Germany	1.13	1.13	1.13	0.92	0.03	0.03	0.03
Canada	1.12	1.12	1.12	0.91	0.03	0.03	0.03
Netherlands	1.09	1.09	1.09	0.86	0.03	0.03	0.03
United Kingdom	1.08	1.08	1.08	0.87	0.02	0.02	0.02
Denmark	1.08	1.08	1.08	0.87	0.02	0.02	0.02

**Table 2:** Variations of the estimated time-varying reproduction number of the epidemic curves and their uncertainty of the Beta variant

Country	R	CI lower	CI upper	R squared	Growth rate	Growth rate CI lower	Growth rate CI upper
Belgium	1.20	1.19	1.21	0.95	0.05	0.05	0.05
Israel	1.17	1.16	1.19	0.97	0.05	0.04	0.05
United States	1.15	1.15	1.16	0.97	0.04	0.04	0.04
Denmark	1.10	1.09	1.11	0.92	0.03	0.02	0.03
Spain	1.10	1.10	1.10	0.72	0.03	0.03	0.03
Germany	1.09	1.09	1.09	0.84	0.03	0.02	0.03
South Africa	1.09	1.09	1.09	0.98	0.02	0.02	0.03
United Kingdom	1.04	1.04	1.04	0.73	0.01	0.01	0.01
Australia	1.04	1.03	1.04	0.35	0.01	0.01	0.01
Botswana	1.03	1.02	1.03	0.24	0.01	0.01	0.01

**Table 3:** Variations of the estimated time-varying reproduction number of the epidemic curves and their uncertainty of the Gamma variant

Country	R	CI lower	CI upper	R squared	Growth rate	Growth rate CI lower	Growth rate CI upper
United States	1.21	1.21	1.21	0.94	0.06	0.06	0.06
Mexico	1.21	1.20	1.21	1.00	0.05	0.05	0.06
Luxembourg	1.17	1.16	1.17	0.70	0.04	0.04	0.05
Canada	1.15	1.15	1.15	0.84	0.04	0.04	0.04
Argentina	1.11	1.11	1.11	0.90	0.03	0.03	0.03
Chile	1.09	1.09	1.09	0.89	0.02	0.02	0.02
Belgium	1.07	1.07	1.07	0.85	0.02	0.02	0.02
Brazil	1.06	1.06	1.06	0.86	0.02	0.02	0.02
Peru	1.05	1.05	1.06	0.94	0.02	0.01	0.02
Italy	1.03	1.03	1.03	0.57	0.01	0.01	0.01

**Table 4:** Variations of the estimated time-varying reproduction number of the epidemic curves and their uncertainty of the Delta variant

Country	R	CI lower	CI upper	R squared	Growth rate	Growth rate CI lower	Growth rate CI upper
France	1.38	1.38	1.38	0.98	0.09	0.09	0.10
India	1.22	1.22	1.22	0.96	0.06	0.06	0.06
Turkey	1.21	1.21	1.22	0.88	0.06	0.06	0.06
Switzerland	1.21	1.21	1.21	0.96	0.05	0.05	0.05
Japan	1.21	1.21	1.21	0.99	0.05	0.05	0.05
United States	1.17	1.17	1.17	1.00	0.05	0.05	0.05
Canada	1.16	1.16	1.16	0.95	0.04	0.04	0.04
Germany	1.14	1.14	1.14	0.99	0.04	0.04	0.04
Denmark	1.04	1.04	1.04	0.68	0.01	0.01	0.01
United Kingdom	1.03	1.03	1.03	0.76	0.01	0.01	0.01

**Table 5:** Variations of the estimated time-varying reproduction number of the epidemic curves and their uncertainty of the Omicron variant

Country	R	CI lower	CI upper	R squared	Growth rate	Growth rate CI lower	Growth rate CI upper
South Africa	1.91	1.87	1.95	0.96	0.20	0.19	0.20
Spain	1.73	1.57	1.91	0.86	0.16	0.13	0.20
Israel	1.63	1.50	1.77	0.92	0.15	0.12	0.17
Denmark	1.63	1.54	1.73	0.92	0.15	0.13	0.16
Belgium	1.63	1.51	1.76	0.92	0.15	0.12	0.17
United States	1.53	1.46	1.61	0.94	0.13	0.11	0.14
United Kingdom	1.52	1.50	1.55	0.94	0.12	0.12	0.13
Australia	1.44	1.31	1.58	0.96	0.11	0.08	0.14
Germany	1.29	1.21	1.37	0.98	0.07	0.06	0.09
Botswana	1.28	1.24	1.32	0.91	0.07	0.06	0.08

**Fig. 6:**
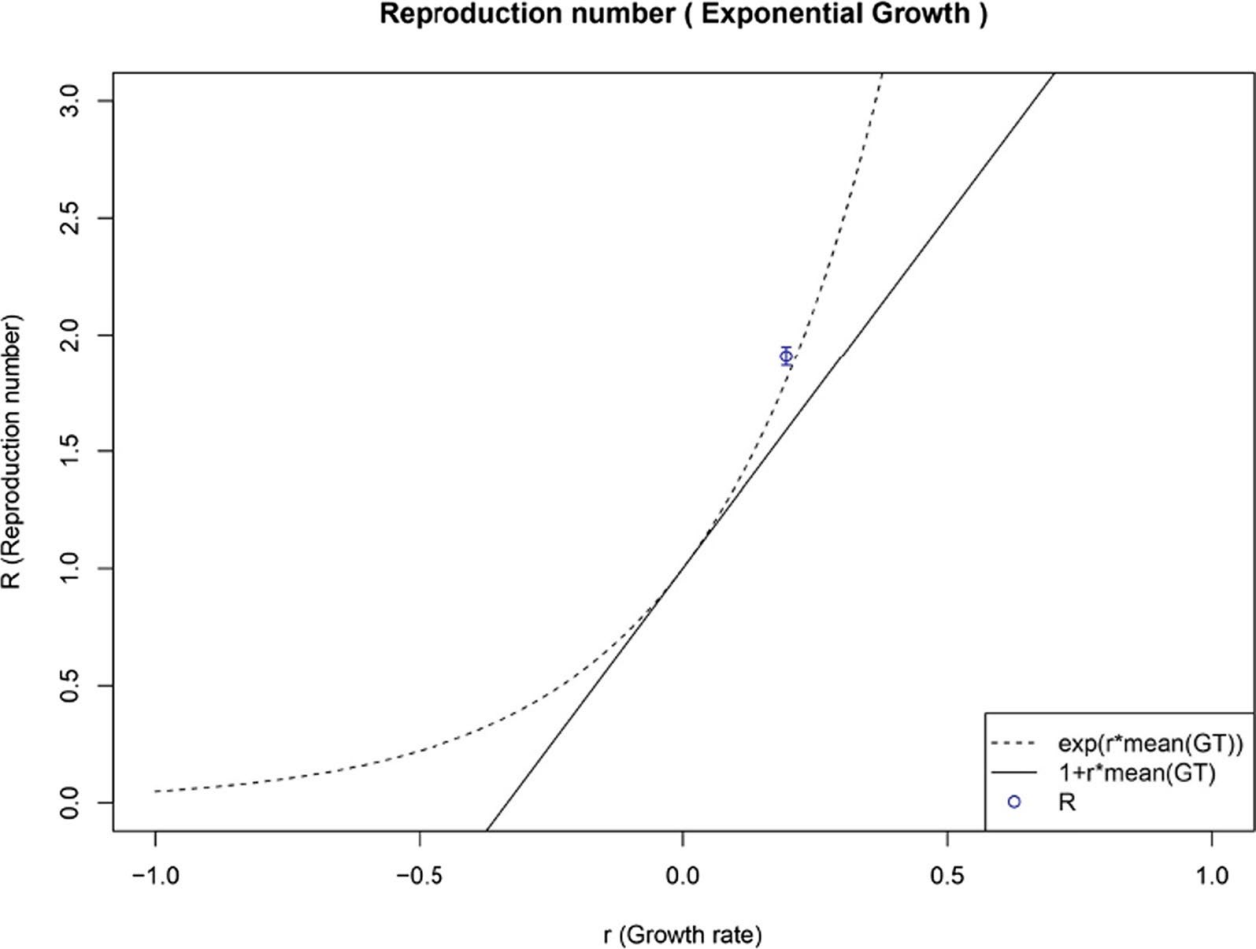
The growth rate (0.195) and the basic reproduction number (1.90) of the Omicron variant reported from South Africa

A growth rate as high as 0.195 was observed in South Africa for Omicron, which calculates into a basic reproduction number of 1.90, as depicted below in Fig. 6.

All the observed epidemic curves had a reproduction number of more than one and a growth rate above 0.007. The highest average reproduction number of 1.559 (sd 0.192) was observed with the Omicron variant, with an average growth rate of 0.130 (sd 0.038). The average R0 and average growth rate of each variant are summarized in Table 6.

**Table 6:** Summary of average R0 and average growth rate of each variant

Variant	Average R0	SD	Average growth rate	SD
Omicron	1.559152	0.192102	0.130183	0.038365
Delta	1.17749	0.098219	0.04649	0.024341
Alpha	1.143135	0.05101	0.038346	0.013009
Gamma	1.114639	0.065154	0.030847	0.01687
Beta	1.101365	0.059286	0.027425	0.015532

The countries with the highest R0 and the variant responsible are summarized in Table 7.

**Table 7:** The countries with the highest R0 in descending order along with the responsible variant

Location	R0	Variant
South Africa	1.907035	Omicron
Spain	1.732674	Omicron
Israel	1.630629	Omicron
Denmark	1.630616	Omicron
Belgium	1.626538	Omicron
United States	1.534041	Omicron
United Kingdom	1.519815	Omicron
Australia	1.438724	Omicron
France	1.380319	Delta
Germany	1.289706	Omicron

## Discussion

The estimated basic reproduction number of the SARS-CoV-2 differ across studies. The WHO initially estimated the R0 as between 1.4 and 2.4. However, the results published are diverse with multiple ranges. Liu et al. reported an R0 range as much as 1.5–6.6 in February 2020. R0 for India was reported as 2.56; China reported an R0 of 2.2, and Italy as 2.4–3.1 [11–13]. It is noted that when stratified by variant, the estimation is understandably lower than that of ‘un-stratified’ R0. Despite stratification, some VOCs have a considerably high growth rate.

The highest R0 for the variants Alpha (1.22), Beta (1.19), Gamma (1.21), Delta (1.38) and Omicron (1.90) were reported from Japan, Belgium, the United States, France and South Africa, respectively. Nine out of ten epidemic curves with the highest estimated growth rates and reproduction numbers were due to the Omicron variant indicating the highest transmissibility followed by Delta, Alpha, Gamma and Beta, respectively.

A limitation of this study is that only a fraction of cases is subjected to sequencing with regard to the Omicron variant. However, the GISAID database encompasses data from 194 countries with over 5 million genetic sequences of SARS-CoV-2 and remains one of the most comprehensive sources of data on SARS-CoV-2 variants [14].

Furthermore, a study published on the Omicron variant noted that by January 2022, more than 200 000 sequences were detected in more than 100 countries suggesting high transmissibility. A Bayesian statistical model incorporating this sequencing data estimated effective reproduction numbers of VOCs yielding similar results to our study [15]. It should be noted that these metrics of transmissibility do not measure the virulence of the variants or their direct impact on global public health but may help gain insight into their pattern of spread.

## Conclusion

This study indicates that the Omicron variant exhibits the highest transmissibility followed by Delta, Alpha, Gamma and Beta respectively.

## Data Availability

Data utilized for this analysis is publicly available at covariants.org and GISAID initiative databases.

## Abbreviations

WHO: World Health Organization
VOI: Variants of interest
VOC: Variants of concern
COVID-19: Coronavirus disease
R0: Basic reproduction number
GISAID: Global initiative on sharing all influenza data
LOESS: Local regression
